# Symptomatic Treatment of Extrapyramidal Hyperkinetic Movement Disorders

**DOI:** 10.2174/1570159X22666240517161444

**Published:** 2024-05-20

**Authors:** Gregory de Boer, Robertus Maria Alfonsius de Bie, Bart Erik Kris Sylvain Swinnen

**Affiliations:** 1 Department of Neurology and Clinical Neurophysiology, Amsterdam UMC Location University of Amsterdam, Meibergdreef 9, Amsterdam, The Netherlands;; 2 Department of Neurology and Neurosurgery, University Medical Center Utrecht, Heidelberglaan 100, Utrecht, The Netherlands

**Keywords:** Extrapyramidal, hyperkinetic, movement disorders, treatment, neuropharmacology, medication, botulinum toxin, deep brain stimulation

## Abstract

Extrapyramidal hyperkinetic movement disorders comprise a broad range of phenotypic phenomena, including chorea, dystonia, and tics. Treatment is generally challenging and individualized, given the overlapping phenomenology, limited evidence regarding efficacy, and concerns regarding the tolerability and safety of most treatments. Over the past decade, the treatment has become even more intricate due to advancements in the field of deep brain stimulation as well as optimized dopamine-depleting agents. Here, we review the current evidence for treatment modalities of extrapyramidal hyperkinetic movement disorders and provide a comprehensive and practical overview to aid the choice of therapy. Mechanism of action and practical intricacies of each treatment modality are discussed, focusing on dosing and adverse effect management. Finally, future therapeutic developments are also discussed.

## 1 INTRODUCTION

Hyperkinetic movement disorders are characterized by excessive, involuntary movements and may greatly impair patients’ daily functioning. Adequate and timely treatment is pivotal. It is, however, a challenge to see the forest for the trees, given the relative rarity and complex phenomenology of these disorders, physicians’ unfamiliarity with the broad range of treatment options, and fear of adverse effects. This generates a reluctance to initiate or increase potentially effective and life-changing treatments.

The objective of this review is to familiarize physicians with the therapeutic options, practicalities and safety of treatments for hyperkinetic movement disorders. To this end, we provide a comprehensive overview of the therapeutic decision tree per phenomenology and discuss practical intricacies (*e.g.*, pharmacological mechanisms, dosing, and adverse effect management) of the different therapeutical classes. This review covers extrapyramidal hyperkinetic movement disorders only (*i.e.*, chorea, ballism, dystonia, tics, and tardive syndromes), as these exhibit a considerable pathophysiological overlap. Hyperkinetic movement disorders with pathology mostly residing outside the basal ganglia (*e.g.,* myoclonus, tremor and hemifacial spasms) will not be covered. Besides medication, this review also describes the role of deep brain stimulation (DBS) and botulinum neurotoxin (BoNT) in the treatment of extrapyramidal hyperkinetic movement disorders. Causal treatments and supportive treatments are not described in this review.

This narrative review may be used as a practical tool for a rational and stepwise treatment of extrapyramidal hyperkinetic movement disorders.

## 2 SEARCH STRATEGY

A literature search was performed on Nov 11, 2022, to identify relevant literature for this narrative review. For this search, PubMed and the Cochrane Database of Systematic Reviews were used. Disease-related topics were “movement disorders”, “dystonia”, “ballism”, “chorea”, “Huntington’s disease”, “tardive syndromes”, “tic disorders”, and “Tourette syndrome”. Treatment-related topics were “treatment”, “botulinum”, and “deep brain stimulation.” Search terms and results are reported in Supplementary Table **S1**. Two authors (G.dB and B.E.K.S.S.) reviewed the titles and abstracts to select relevant articles. Additionally, relevant articles were identified by snowballing using the references in selected articles.

## 3 TREATMENT OF HYPERKINETIC MOVEMENT DISORDERS BY PHENOMENOLOGY

### 3.1 Chorea

Chorea is defined as irregular, random, brief, sudden, rapid, jerky, unsustained movements [1]. Chorea occurs in several neurodegenerative disorders (*e.g.*, Huntington’s disease, neuroacanthocytosis, neurodegeneration with brain iron accumulation, and certain types of spinocerebellar ataxia), autoimmune disorders (*e.g.*, Sydenham’s chorea and systemic lupus erythematosus), structural brain lesions, paroxysmal movement disorders, and medication-induced movement disorders (*e.g.*, tardive dyskinesias and levodopa-induced dyskinesias) [2]. The pathophysiological processes responsible for the induction of chorea are poorly understood, but a hyperdopaminergic and/or hyperglutamatergic state is suspected [3].

**Fig. (1):**
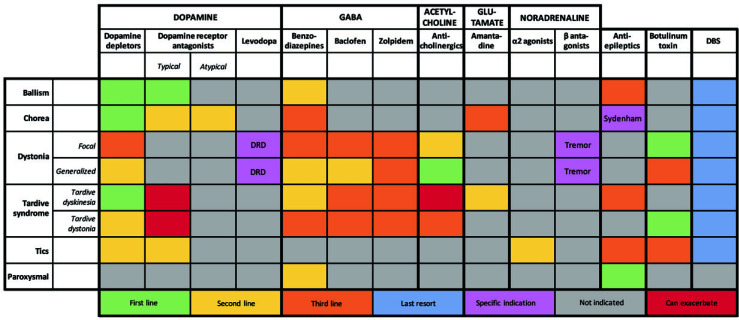
Heat map of extrapyramidal hyperkinetic movement disorder subtypes and therapeutic approaches. For each extrapyramidal movement disorder subtype therapeutic options are depicted, stratified by neurotransmitter system and/or pharmacological class. Color (legend see bottom of the heat map) indicates the position of each treatment in the respective therapeutic tree according to the authors’ opinion, which is based on available literature and experience. **Abbreviations**: DBS, deep brain stimulation; DRD, dopa-responsive dystonia; GABA, gamma-aminobutyric acid.

Dopamine-depleting agents (DDAs) are considered the treatment of choice for most chorea, given their effectivity, broad applicability, and favorable adverse effects profile (Fig. **1** and Table **1**). Their efficacy in Huntington's disease has been demonstrated in several randomized controlled trials (RCTs), whereas their use in other causes of chorea mostly relies on observational studies, case series, or case reports [4, 5].

Dopamine receptor blocking agents (DRBAs) may be preferred in cases with associated psychotic symptoms, acute severe chorea, or contraindications for DDAs [5, 6]. Otherwise, DRBAs are considered second-line treatment after DDAs, mainly because of the risk of tardive syndromes [5]. While in clinical practice, basically all typical DRBAs are used for chorea treatment, only the use of tiapride is supported by an RCT [5]. True atypical DRBAs (*i.e.*, quetiapine and clozapine) can also be employed, but evidence regarding quetiapine is weak, and the adverse effects profile of clozapine is unfavorable [5].

Antiglutamatergic agents are less suited for the treatment of chorea, given their limited efficacy [5]. However, in the context of Parkinson’s disease, amantadine was shown to be an effective treatment for levodopa-induced dyskinesias [7]. For other forms of chorea, amantadine can occasionally be helpful as an adjunctive drug in individual cases, whereas riluzole is not indicated [8]. Similarly, agents acting on the neurotransmitter gamma-aminobutyric acid (GABA) can be of occasional limited use, but solid evidence is not available [5].

For some types of chorea, it may be possible to tailor symptomatic treatment; valproic acid is helpful in Sydenham’s chorea and chorea due to structural lesions, carbamazepine (and other antiepileptic drugs) is indicated for paroxysmal movement disorders, and levodopa-induced dyskinesias primarily require adjustment of the dopaminergic medication regime [5].

In refractory cases of chorea, deep brain stimulation (DBS) can be considered. In Huntington’s disease, DBS targeting the globus pallidus internus (GPi) significantly reduces chorea but without clear improvement in quality of life, mostly due to its lack of effect on other (motor) problems and its tendency for stimulation-induced dystonia and bradykinesia [9]. Although not evidence-based, in individual cases, DBS might be a last resort treatment for any other cause of severe refractory chorea.

### 3.2 Ballism

Ballism has the same characteristics as chorea, but movements are flinging, are more proximal, and have a larger amplitude. It usually occurs unilaterally (hemiballism) and is mostly caused by a structural brain lesion [10]. Ballism is to be regarded as an extreme (proximal) form of chorea [11]. Hence, the treatment of ballism and chorea are very much alike. However, given the acute and sometimes life-threatening nature, potent drugs with immediate onset of action, like typical DRBAs, are generally used (Fig. **1**). In cases that are not life-threatening or debilitating (anymore), DDAs are preferred, given their favorable adverse effects profile for long-term use. Benzodiazepines (mostly clonazepam) can be useful in the acute setting and have an additive sedating and muscle-relaxing effect [12]. Similarly, certain antiepileptic drugs (*e.g.*, valproic acid and topiramate) can have some adjunctive benefits [12]. In very severe refractory ballism, GPi-DBS might be the last resort [13].

### 3.3 Dystonia

Dystonia is characterized by sustained or intermittent muscle contractions causing abnormal, often repetitive, movements, postures, or both [14]. Dystonia generally presents as an idiopathic isolated syndrome (*e.g.*, cervical dystonia), but can also be a manifestation of hereditary disorders, idiopathic neurodegenerative disorders (*e.g.*, Parkinson’s disease and progressive supranuclear palsy), a structural brain lesion, or medication-induced movement disorders (*e.g.*, levodopa-induced dystonia and tardive dystonia) [15]. The exact mechanism leading up to dystonic movements is still unclear, but most probably concerns a circuitopathy with involvement of cholinergic, dopaminergic and GABAergic signaling [15]. Therefore, drugs targeting these neurotransmitters are implicated in the management of dystonia (Fig. **1**).

Focal and segmental dystonia are mainly treated with botulinum neurotoxin (BoNT) injection of the overactive dystonic muscles [16]. This preference resides in its efficacy, broad applicability, and lack of systemic adverse effects [16]. Anticholinergics (mainly trihexyphenidyl) are indicated when BoNT is insufficient, contraindicated (*e.g.*, due to technical or anatomical issues), or not tolerated [17]. Anticholinergics are generally less effective and less tolerated than BoNT [17]. DDAs and GABAergic medication (*i.e.*, benzodiazepines, muscle relaxants, and anti-spasticity agents) can have some effect as an add-on treatment [18]. Treatment of generalized dystonia is very parallel to focal dystonia, with the important difference that BoNT is of less use given the extensiveness of the dystonia [15]. In selected cases, however, injection of the most affected muscles can be considered. In severe refractory cases of focal or generalized dystonia, DBS targeting the GPi (but also the subthalamic nucleus) is a valid option [15]. The effect of DBS is variable but often leads to a significant improvement, the extensiveness of which generally depends on the underlying disease, with the best effects seen in idiopathic and/or isolated dystonia [19].

Some forms of dystonia allow or require specific treatment. Dopa-responsive dystonias (*e.g.*, due to *GCH1* or *AADC* mutation) respond very well to low doses of levodopa and/or dopamine agonists, Wilson’s disease requires copper targeting therapies, and paroxysmal movement disorders may respond to anti-epileptics, acetazolamide, or ketogenic diet [15]. Moreover, when the clinical picture is dominated by (dystonic) tremor, beta-antagonists or other tremor-targeted treatments can be useful.

### 3.4 Tics

Tics are sudden, rapid, recurrent, nonrhythmic, and purposeless movements or vocalizations [20]. Tics are preceded by a premonitory urge that is relieved by tic performance and can temporarily be suppressed. Tics mainly occur in the setting of Tourette syndrome and are then generally accompanied by psychiatric and/or behavioral symptoms (*e.g.*, obsessive-compulsive disorder (OCD) and attention-deficit hyperactivity disorder (ADHD)) [21]. Tics can also be secondary to medication (*e.g.*, DRBAs), toxins (*e.g.*, cocaine and amphetamines), neurodevelopmental disorders (*e.g.*, autism spectrum disorder), post-infectious and auto-immune neurological disorders (*e.g.*, pediatric autoimmune neuropsychiatric disorders associated with streptococcus, and anti-LGI1 encephalitis), and neurodegenerative disorders (*e.g.*, Huntington’s disease, neuro-acanthocytosis, and neurodegeneration with brain iron accumulation) [22, 23]. A secondary cause should be suspected in adult-onset tics or the presence of other (dominating) neurological features. The underlying mechanism of tics is poorly understood but probably involves several neurotransmitter systems (*e.g.*, dopamine, noradrenaline, and GABA) [24].

Whereas treatment of tics in Tourette syndrome has extensively been investigated, even resulting in useful clinical guidelines, treatment of secondary tics is largely uncharted and relies on case reports and extrapolation from Tourette management [25, 26]. Management of secondary tics is generally in line with Tourette syndrome, except for α2 adrenergic agonists being ineffective and DDAs being the treatment of choice for tardive tics.

Importantly, tics often do not require tic-specific treatment, mostly because psychiatric and/or neurological comorbidities dominate the clinical picture but also because patients are often not too distressed about their tics. In case tics are mild but still perceived as bothersome, psycho-education to improve coping may suffice.

The first line of actual tic treatment is a non-pharmacological approach constituting cognitive-behavioral treatment. This includes comprehensive behavioral intervention for tics - encompassing habit reversal training and competing response training - and exposure response prevention [23]. Cognitive-behavioral treatment is probably insufficiently effective for very severe to malignant tics, in which case immediate second-line treatment is warranted.

Therapy with DRBAs, DDAs, and α2 adrenergic agonists constitutes the second line of tic treatment but should only be initiated when tic severity and burden outweigh the risks of pharmacological treatment (Fig. **1**). The choice of product is inspired by the tic-phenotype (*e.g.*, location and severity), comorbidities (*e.g.*, psychiatric and metabolic), and the product’s adverse effect profile. Whereas the efficacy of several typical DRBAs, like tiapride, risperidone, haloperidol, and pimozide, is supported by several RCTs, aripiprazole is often preferred because of the somewhat better adverse effect profile [26]. Nevertheless, DRBA use unavoidably implies a substantial risk of adverse effects, with permanent extrapyramidal adverse effects being the most troublesome. The atypical antipsychotics clozapine and quetiapine are insufficiently effective but have only rarely been investigated [27]. Therefore, DDAs may be an appropriate alternative second-line agent. However, RCTs assessing deutetrabenazine and valbenazine were negative, and tetrabenazine has not been investigated in an RCT [28]. Tetrabenazine use is, however, supported by several non-controlled studies [29]. DDAs are not mentioned in current guidelines, despite DDA use constituting a prerequisite to consider DBS [25, 26]. Since α2 adrenergic agents guanfacine and clonidine improve ADHD symptoms, these are good second-line options for Tourette patients with ADHD comorbidity but otherwise have limited effect on tics [24, 26].

Topiramate has a limited effect size and is often not tolerated [25]. Lamotrigine is not advised as it may worsen tic disorders [30, 31]. BoNT injections are a valid option for medication-refractory, focal, simple tics, especially when self-injurious [25].

DBS can be considered for severe medication-refractory cases and has currently been performed in over 300 patients worldwide [21]. Whereas DBS for tics is supported by a prospective registry and one RCT, there is a lack of well-designed and sufficiently powered RCTs [25, 32, 33]. Selection criteria are still under debate, mostly regarding age limit, disease duration, and presence of significant comorbid psychiatric issues [9]. The optimal target is also unclear, but the centromedian thalamus and GPi seem the most effective [32].

### 3.5 Tardive Syndromes

Tardive hyperkinetic movement disorders can be caused by any drug with an affinity for the D2 receptor, mainly constituting DRBAs like antipsychotics, metoclopramide, and sulpiride. A pleiotropy of other agents (*e.g.*, calcium blocking agents, anti-epileptics, lithium, antihistaminic agents, and chloroquine) also has some D2 receptor affinity and hence can infrequently cause tardive syndromes. Tardive syndromes typically emerge late in the course of treatment and often persist despite discontinuation of the offending agent [20]. The underlying pathophysiology remains elusive but mainly involves D2 receptor hypersensitivity and probably also some degree of maladaptive plasticity involving several neurotransmitter systems (*i.e.*, glutamate, GABA, and acetylcholine) [34, 35].

Tardive hyperkinetic syndromes display a huge phenomenological variability. Oro-bucco-lingual choreatiform hyperkinesia, coined tardive dyskinesia, is the most frequent phenotype. Tardive dystonia, mainly involving the facial-craniocervical region, is the second most common presentation. Other tardive syndromes (*e.g.*, tic, chorea, tremor, or myoclonus) are infrequent, with limited data regarding their treatment. Hence, we here focus on tardive dyskinesia and tardive dystonia.

Discontinuing the offending agent is inarguably the first and intuitive step in tardive syndrome management, which is straightforward for many causative agents but not for antipsychotics. Substituting or discontinuing antipsychotics is often clinically unfeasible for psychiatric reasons. Taking into consideration that tardive syndromes are irreversible despite discontinuation of the offending agent in up to 87% of cases and that there is no solid evidence supporting this approach, discontinuing antipsychotic treatment may not be necessary nor useful after all [36-39]. While the jury is still out regarding antipsychotic discontinuation, there are some non-controlled studies in support of switching the antipsychotic to clozapine in case of a moderate to severe tardive syndrome [37, 39, 40]. Despite an initial beneficial effect, initiating a typical antipsychotic to improve a tardive syndrome caused by a non-antipsychotic is definitely contraindicated because this strategy implies a continuation and aggravation of the underlying problem in the long term [39].

The first-line treatment of tardive dyskinesia constitutes DDAs, with deutetrabenazine and valbenazine exhibiting solid RCT evidence (Fig. **1**) [39, 41, 42]. In tardive dystonia, especially focal dystonia, BoNT injections are probably the first choice, which is supported by several mostly retrospective uncontrolled studies and extrapolation from idiopathic dystonia [34]. There is no good data to support BoNT for tardive dyskinesia [34].

When DDAs for tardive dyskinesia are ineffective or not tolerated, clonazepam and amantadine can be considered, which are both supported by one small RCT [34, 39, 43, 44]. When BoNT for tardive dystonia is not effective, not tolerated, or impossible, DDAs should be considered next.

Baclofen, zolpidem, and levetiracetam can be considered as the third line for tardive dyskinesia, but these options lack solid evidence and have limited effect [34]. Similarly, and with the same side note, clonazepam, baclofen, zolpidem, and anticholinergics can be considered for tardive dystonia [34, 38, 39, 45]. Anticholinergics may, however, exacerbate tardive dyskinesia [39, 45, 46].

When severe and medication-refractory, DBS targeting the GPi can be considered for tardive syndromes. Several uncontrolled studies, collectively encompassing 117 patients, demonstrate good to often excellent effects [47, 48]. However, publication bias is likely, which may be corroborated by the one small RCT being negative [49, 50]. Moreover, since most of the 117 patients had tardive dystonia and only four had tardive dyskinesia, the effectivity of DBS in the latter is most unclear [48].

## 4 THERAPEUTIC ARSENAL FOR EXTRAPYRAMIDAL HYPERKINETIC MOVEMENT DISORDERS

### 4.1 Dopaminergic Medication

#### 4.1.1 Dopamine-depleting Agents (DDAs)

**Fig. (2):**
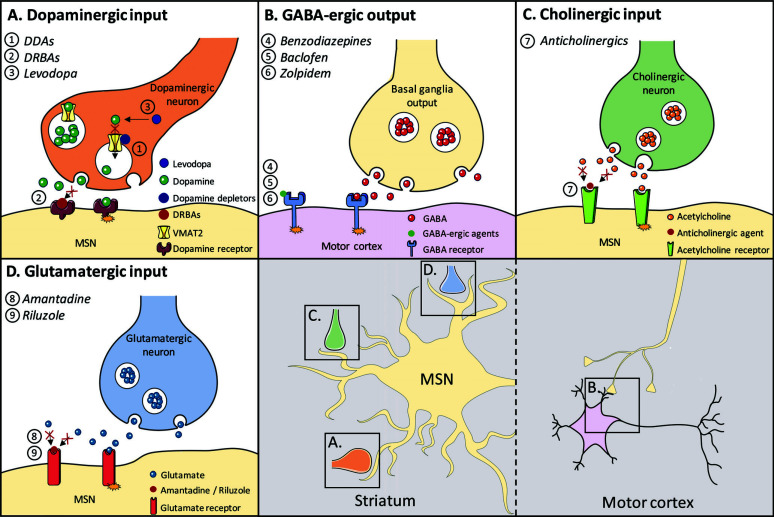
Mechanisms of pharmacological therapies targeting basal ganglia function for hyperkinetic movement disorders. The modes of action of pharmacological agents targeting the dopaminergic, GABA-ergic, cholinergic or glutamatergic neurotransmitter system are depicted. **Abbreviations**: DDAs, dopamine depleting agents; DRBAs, dopamine receptor blocking agents; GABA, gamma-aminobutyric acid; MSN, medium spiny neuron; VMAT2, vesicular monoamine transporter 2. *Color legend of neurons*: yellow, basal ganglia neuron (MSN); purple, motor cortex; orange, dopaminergic neuron; green, cholinergic neuron; blue, glutamatergic neuron.

As their name implies, DDAs deplete presynaptic vesicles from dopamine. All currently marketed DDAs target the vesicular monoamine transporter 2 (VMAT2), which loads presynaptic vesicles with monoaminergic neurotransmitters (*e.g.,* dopamine, noradrenaline, and serotonin - Fig. **2**). Hence, by decreasing dopamine levels in presynaptic terminals, DDAs accomplish a relative depletion of dopamine in the synaptic cleft, decreasing dopaminergic signaling [51].

Three selective VMAT2 inhibitors are currently used to treat extrapyramidal hyperkinetic movement disorders: tetrabenazine, deutetrabenazine, and valbenazine. Deutetrabenazine and valbenazine have a longer half-life than tetrabenazine, and therefore, once-daily dosing suffices, more stable plasma levels are reached, which explains their improved adverse effects profile [52]. Possible dosing schemes are suggested in Table **2**. DDAs all concern prodrugs that are metabolized into active metabolites [51]. DDAs are metabolized by the cytochrome P450 through CYP2D6. In patients treated with a tetrabenazine dose of 50 mg a day or higher, CYP2D6 genotyping can be considered, as intermediate and poor metabolizers have a higher risk of adverse effects [53, 54]. A maximum dosage of 100 mg a day is advised. In deutetrabenazine, a maximum dosage of 36 mg a day is recommended in CYP2D6-poor metabolizers [55]. Caution is advised in patients using already a strong CYP2D6 inhibitor (*e.g.,* fluoxetine, paroxetine). Besides CYP2D6, valbenazine is also metabolized by CYP3A4. Concomitant use of a CYP3A4 inducer (*e.g.,* rifampicin, carbamazepine, phenytoin) is not recommended. A maximum dose of 40 mg a day is recommended in patients who also use a CYP3A4 inhibitor (*e.g.,* itraconazole, clarithromycin) [56].

The most important and most feared adverse effects of DDAs are depression, sedation, and parkinsonism, related to the depletion of serotonin, noradrenaline, and dopamine, respectively. These also constitute the major relative contra-indications for DDA use. Suicidality is considered an absolute contra-indication [57]. Adverse effects are dose-dependent and can be reduced by slowly titrating or by decreasing the dosage [58]. Other strategies to manage adverse effects are listed in Table **1**. For example, the first step in the management of depression in patients using a DDA is dose reduction, and initiating antidepressants can be a second step. With drug-induced parkinsonism, the first step is reducing the dosage. A second step can be the addition of an anticholinergic drug like trihexyphenidyl in patients under 60 years of age or amantadine in patients over 60 years of age. The third step can be adding levodopa [59, 60]. It is important to evaluate whether another type of agent is preferred in patients experiencing significant adverse effects. The major advantage of DDAs over DRBAs lies in their lack of inducing tardive syndromes [61]. Iatrogenic induction of a potentially disabling and persistent movement disorder in a patient already suffering from a hyperkinetic movement disorder is against the general dogma *primum nonnocere*. It needs to be prevented at all costs. Therefore, in many instances, DDAs should be preferred over DRBAs.

#### 4.1.2 Dopamine Receptor Blocking Agents (DRBAs)

DRBAs (or neuroleptics or antipsychotics) bind to the postsynaptic dopamine D2 receptor and interfere with the binding of dopamine to the receptor, hence decreasing dopaminergic signaling (Fig. **2**) [62]. There is a variation in receptor binding affinity and selectivity between different DRBAs, which explains their different effectivity and adverse effects profile [63]. For example, a higher affinity for the D2 receptor results in a higher potency, leading to a higher risk of extrapyramidal adverse effects [62, 63]. Besides D2 receptor binding, DRBAs also bind to histamine, muscarinic and alpha-adrenergic receptors.

DRBAs can be divided into typical and atypical DRBAs, and this division is mainly based on receptor affinity. Atypical DRBAs pharmacologically act as a major 5-HT2A (serotonin) antagonist and have a lower D2 affinity compared to typical antipsychotics [63]. Clozapine and quetiapine are the ones with the least D2 receptor affinity and are to be considered the only true atypical DRBAs.

Generally used dosages are described in Table **2**. One of the main groups of adverse effects of the D2 receptor effect of antipsychotics is extrapyramidal adverse effects (*e.g.,* tardive dyskinesia or dystonia, akathisia, and parkinsonism) [64]. Clozapine and quetiapine have the lowest rates of extrapyramidal adverse effects [63]. There are different strategies for treating specific adverse effects (Table **1**). The first step advised in tardive syndromes is ceasing the causative agent if feasible. Slowly reducing the dosage is advised, as a quick withdrawal can lead to withdrawal symptoms. If DRBAs are the only therapeutic option, it is advised to switch to clozapine or quetiapine because of lower D2 receptor affinity. Other options for treating tardive syndromes are described in the section on tardive syndromes.

Other common adverse effects mostly reside in non-D2 receptor affinities, encompassing sedation, hypotension, weight gain, and metabolic syndrome [61, 63]. A rare but important idiosyncratic adverse effect is neuroleptic malignant syndrome. The offending agent needs to be discontinued immediately, and supportive treatment should be initiated. Dantrolene, bromocriptine, and lorazepam can be considered in severe cases. In refractory cases, electroconvulsive therapy may be a last resort treatment [65, 66]. Usefulness of clozapine is limited by several adverse effects. Besides sedation and weight gain, clozapine use implies a small risk of life-threatening agranulocytosis, which requires close monitoring of leukocyte values [67]. Clozapine can also induce myoclonus or seizures [68, 69]. Clozapine-induced myoclonus can occur at low plasma levels and can precede tonic-clonic seizures [69]. When seizures occur without myoclonus, the first treatment option is reducing clozapine dosage [68]. Advised treatment of seizures in clozapine-induced myoclonus is valproic acid if reducing the clozapine dosage is not feasible [69]. These clozapine-specific adverse effects result in quetiapine having the most favorable adverse effects profile among the available DRBAs. Aripiprazole is also considered to have a slightly better adverse effects profile than other DRBAs [31].

### 4.2 GABAergic Medication

GABA is the main inhibitory neurotransmitter in the central nervous system. GABA is produced in the presynaptic neuron by conversion of glutamate by glutamic acid decarboxylase. GABA binds to the postsynaptic GABAa and GABAb receptors. GABAergic medications such as benzodiazepines, baclofen, and zolpidem increase the activity of GABA [70].

Benzodiazepines (*e.g.,* clonazepam, diazepam, alprazolam) act on the GABAa receptor by binding to the *alpha* and *gamma* subunits. This increases the postsynaptic receptor’s affinity for GABA and enhances GABAergic transmission [71].

Adverse effects of benzodiazepines are dose-dependent, with sedation, fatigue, and impaired concentration being the most frequent. Caution with prescribing benzodiazepines is even more important in the elderly, with an increased risk of falls, paradoxical reactions, and amnesia [72]. Dependence on benzodiazepines occurs in up to half of patients [73]. An effect of benzodiazepines on dopaminergic neurons in the ventral tegmental area has been suspected to mediate this addiction [72]. Doses differ between different benzodiazepines; advised doses of clonazepam - the most widely used benzodiazepine in hyperkinetic extrapyramidal movement disorders - can be found in Table **2**.

Baclofen is a GABA-B receptor agonist that reduces the release of excitatory neurotransmitters in the brain and the spinal cord [74]. It can be administered orally in most disorders. However, intrathecal administration is often employed in patients with dystonia secondary to cerebral palsy. Effective oral dosages range from 30 to 120 mg daily, divided over 3 to 4 doses [67]. Common adverse effects include sedation, nausea, impaired mentation, dizziness, and loss of muscle tone [74, 75].

Zolpidem is a GABAa receptor agonist acting more selectively on the *alpha1* subunits, resulting in less anxiolytic and cognitive adverse effects than benzodiazepines [76, 77]. Possible adverse effects are sedation, cognitive impairment, ataxia, and depression. Zolpidem is associated with a lower dependence rate compared to benzodiazepines [76].

In general, titrating GABAergic medication slowly is advised to prevent the occurrence of adverse effects. If adverse effects are mild to moderate, dose holding or dose reduction can be advised. Sudden discontinuation is to be avoided for all GABAergic agents, as this can lead to withdrawal symptoms, including seizures [72, 76]. Gradual discontinuation over weeks or months, with concomitant psychotherapy if appropriate, is advised to prevent withdrawal symptoms [72, 76, 78].

### 4.3 Anticholinergics

Anticholinergic agents inhibit the activity of the neurotransmitter acetylcholine (ACh) by blocking the ACh receptors at both central and peripheral nervous system synapses [79, 80]. Cholinergic receptors can be classified into nicotinic and muscarinic receptors. Nicotinic receptors are present in the central and peripheral nervous system and muscle, among other tissues. In this review, we will focus on agents mainly blocking the muscarinic ACh receptors in the basal ganglia [67, 81].

The main anticholinergic agent used in extrapyramidal hyperkinetic movement disorders is trihexyphenidyl [82]. However, biperiden, benztropine, or other agents have been used in some cases [67, 82]. Trihexyphenidyl and biperiden are both muscarinic Ach-receptor antagonists. Both agents also have a dopaminergic effect. However, the exact physiology is unknown [83].

The recommended starting dose of trihexyphenidyl is 2 mg twice daily and can be increased by 2 mg every few days. The effective dosage is 5-15 mg divided over 3-4 doses per day. Higher dosages have been prescribed but are often accompanied by unbearable adverse effects [67, 75]. Biperiden is generally administered 2 mg 1-3 times a day [84, 85].

Adverse effects relate to the central and peripheral effects of anticholinergic agents. Central adverse effects include confusion, sedation, psychosis, and cognitive impairment [67]. Peripheral adverse effects are dry mouth, constipation, urinary retention, tachycardia, and blurred vision [67, 75]. Dose holding or dose reduction is advised when troubling adverse effects occur. When this is insufficient, treatment of individual symptoms while maintaining the dose can be considered (*e.g.*, beta-blockers for tachycardia or laxative agents for constipation) [86]. In general, anticholinergics are tolerated well by individuals below 60 years of age. Caution with prescribing anticholinergic agents is advised in elderly patients because of the increasing prevalence of cognitive dysfunction, benign prostate hypertrophy, and glaucoma in this population. The use of anticholinergic agents is also associated with an increased frequency of falls in the elderly [67, 82, 87].

### 4.4 Amantadine

Amantadine is a drug with multiple mechanisms of action. It mainly acts as an antagonist on the glutamate NMDA receptor in the putamen and subthalamic nucleus, however it also targets the dopaminergic, cholinergic, and serotonergic systems. Glutamate is the most important excitatory neurotransmitter in the central nervous system and has a major role in neuronal plasticity [88]. In the presynaptic neuron, glutamine is converted to glutamate by glutaminase [89]. There are several postsynaptic glutamate receptors, which include the ionotropic alfa-amino-3-hydroxy-5-methyl-4-isoxazole-propionic acid (AMPA), kainate and N-methyl-D-aspartate (NMDA) receptors, and the metabotropic glutamate receptors [90, 91]. There are multiple glutamatergic drugs used in extrapyramidal hyperkinetic movement disorders, which act on the glutamatergic pathway in different ways [90]. Most of these drugs are used as antiepileptic drugs (AED) and will be discussed in the next chapter. This part only covers amantadine.

The starting dosage of amantadine is 100 mg once daily and is mostly administered 100 mg twice daily. In some cases, it can be dosed at 200 mg twice daily. The dose should be adjusted in patients with impaired renal function [92]. Central nervous adverse effects include agitation, insomnia, seizures, and psychosis [93]. To prevent hallucinations, confusion, and insomnia at night, the last dose should not be taken too late during the daytime [92]. Peripheral adverse effects encompass constipation, dizziness, dry mouth, or nausea [93]. Idiosyncratic adverse effects can be keratopathy or livedo reticularis, both reversible upon amantadine discontinuation [94, 95].

### 4.5 Antiepileptic Drugs

Several AEDs are used in extrapyramidal hyperkinetic movement disorders. Some agents have a specific indication, whereas other drugs are used in multiple movement disorders.

AEDs increase GABAergic and/or decrease glutamatergic signaling by engaging different targets. At the presynaptic level, these comprise synaptic vesicle glycoprotein 2A (*e.g.*, levetiracetam), and voltage-gated sodium (*e.g.,* carbamazepine and valproic acid) and calcium (*e.g.,* gabapentin and pregabalin) channels. On the postsynaptic membrane, these include glutamate (*e.g.,* topiramate and perampanel) and GABA (*e.g.,* topiramate) receptors. Whereas in epilepsy, AEDs are believed to execute their function in the cerebral cortex, AEDs may improve extrapyramidal hyperkinetic movement disorders at the level of the basal ganglia [96-98].

Each AED has a different dosing regimen. In most AEDs, the dose has to be increased stepwise (*e.g.,* lamotrigine and topiramate). General adverse effects of AEDs are sedation, dizziness, and nausea [99, 100]. There are multiple specific adverse effects of AED treatment. Gabapentin and valproic acid can induce weight gain, whereas topiramate can induce weight loss. Visual field defects have been reported in gabapentin, carbamazepine, and phenytoin. Different dermatologic adverse effects are reported in AED use. Whereas rash is a common and benign adverse effect in lamotrigine use, Stevens-Johnson syndrome is a rare but potentially life-threatening adverse event (0.1-0.4%). Concomitant use of valproic acid increases the frequency of dermatological adverse effects. Phenytoin, carbamazepine, and lamotrigine can induce a drug hypersensitivity syndrome, leading to an exanthematous skin reaction and lymphadenopathy. In Stevens-Johnson syndrome and drug hypersensitivity syndrome, the causative agent has to be discontinued immediately. Furthermore, lamotrigine can lead to hepatotoxicity, granulomatous interstitial nephritis, or colitis. Movement disorders caused by AEDs are also described. For example, phenytoin and lamotrigine can induce chorea, and gabapentin can cause asterixis or myoclonus. Psychiatric disorders such as psychosis and depression are associated with topiramate and zonisamide [99]. Dose reduction or discontinuation of the causative drug constitutes the first step in the management of most adverse effects.

### 4.6 Botulinum Neurotoxin

Botulinum neurotoxin (BoNT) is a bacterial neurotoxin produced by *Clostridium botulinum* [75]. It causes muscle weakness by inhibiting the release of acetylcholine into the neuromuscular junction [101]. BoNT reaches maximum effect after 1 to 2 weeks. The duration of the effect is approximately 13 weeks [102, 103].

BoNT has been used in movement disorders since 1981 and has been approved for use in multiple disorders [101, 104]. Different brands are FDA-approved, with some containing BoNT serotype A and others containing BoNT serotype B. Dosing is brand- and patient-specific and is mainly inspired by phenotype, severity, effectivity, and adverse effects. Dose finding generally comprises a trial-and-error approach.

Adverse effects can be divided into three different categories. The first category relates to the primary mechanism of action of BoNT. Temporary weakness in targeted (or neighboring) muscles occurs frequently, leading to neck weakness, ptosis, diplopia, facial asymmetry, or dysphagia, amongst others [105, 106]. Options to prevent and/or manage these adverse effects can be lowering the dose or to inject EMG- or ultrasound-guided to treat only the affected muscles. The second category of adverse effects is injection-related, for example, injection pain or hematoma. To treat or prevent pain, topical anesthetic agents can be used. Hematoma can be treated by applying pressure or cooling the skin with an ice pack [107]. The third category is systemic adverse effects. These are mainly flu-like symptoms consisting of elevated body temperature, myalgia, and malaise [108, 109]. Analgesics and antipyretic drugs can be used when flu-like symptoms occur [110].

Over time, treatment failure can arise because of neutralizing antibodies [104]. The risk of developing neutralizing antibodies increases with increasing BoNT doses, accounting for the higher incidence in, for example, cervical dystonia (around 15%) when compared to blepharospasm (around 5%) [104, 111]. To prevent the development of neutralizing antibodies, BoNT should be dosed as low as possible and intervals between injections should be as long as possible, preferably at least three months [112, 113]. The preferred treatment strategy when neutralizing antibodies occur is uncertain, but encompasses switching to an alternative serotype of BoNT, temporarily discontinuing the treatment with BoNT, switching to medication, or to deep brain stimulation (DBS) [113].

### 4.7 Deep Brain Stimulation

With DBS, continuous electrical stimulation is applied to deep brain structures. DBS has been established as an effective and safe treatment for medication-refractory essential tremor and Parkinson’s disease. DBS is currently also frequently employed in dystonia and is under investigation in practically all extrapyramidal hyperkinetic movement disorders (Fig. **1**) [114]. Different indications have different anatomical targets for DBS (Table **3**), and efficacy is different in specific disorders. The exact mode of action of DBS is still unclear but probably concerns an interruption of pathological neuronal activity - in keeping with previous lesion therapies for movement disorders.

Adverse effects related to DBS are twofold. The surgical procedure and the presence of foreign material pose certain risks. The most feared and bothersome complications are intracranial hemorrhage (1%) and infection (1-9%) [115]. In some cases, intracranial hemorrhage can lead to hemiparalysis. However, most cases of intracranial hemorrhage are asymptomatic [116]. Postoperative infection can be treated with antibiotics in most cases. However, sometimes a second surgery is required [117]. Other complications include periorbital hematoma, delayed peri-lead edema, lead and/or cable fracture, and skin erosion. Next to these surgical and hardware complications, the intended or unintended electrical stimulation of brain regions can lead to several stimulation-induced adverse effects. Evidently, their nature is determined by the lead localization and local anatomy. Hence, stimulation-induced adverse effects differ per target. Fortunately, stimulation-induced adverse effects are transient and can mostly be resolved by DBS parameter adjustments. Stimulation of the ventral intermediate nucleus of the thalamus, posterior subthalamic area, and dentatorubrothalamic tract can lead to sensory (*e.g.,* paresthesia, numbness, dysgeusia) and cerebellar (*e.g.,* ataxia, dysarthria) adverse effects [118]. Stimulation of the subthalamic nucleus can lead to adverse effects on multiple domains, *e.g*., motor (dyskinesia), pyramidal (dysarthria, leg dragging), oculomotor (diplopia), behavioral system (depressed mood, apathy, impulsivity) and cognitive system (decreased cognition) [119]. GPi stimulation can mainly lead to visual (phosphenes), motor (parkinsonism), and pyramidal (dysarthria, leg dragging) adverse effects [116, 120-123].

## 5 FUTURE

Current treatment of extrapyramidal hyperkinetic movement disorders is hampered by several limitations, mainly concerning limited efficacy, unclear efficacy, and/or unfavorable adverse effect profiles. A better understanding of underlying mechanisms is pivotal to propel pharmacological and technological innovations, with subsequent validation in well-designed, sufficiently powered, and preferably head-to-head comparison RCTs.

Some upcoming pharmacological innovations are noteworthy; deutetrabenazine is currently under investigation for the treatment of dystonia, a novel D1 receptor antagonist - ecocipam - may be a promising new treatment for tics, and, albeit clinical trials have been negative so far, cannabinoids may enrich the therapeutic arsenal [30, 124, 125].

Whereas BoNT has been used in movement disorders for decades already, practice and formulations have not changed much ever since. BoNT serotypes with a longer duration (*e.g.,* daxibotulinumtoxinA lasting 24 weeks) could lead to fewer adverse effects, longer duration of action (and hence less frequent injections), and less resistance due to neutralizing antibodies [126]. Radical changes in BoNT injection technique are to be expected in the coming decade. Muscle selection and targeting may be improved by using ultrasound guidance [127]. RCTs comparing ultrasound-guided BoNT injections with electromyography-guided injections in dystonia will, however, be necessary to evaluate the difference in efficacy between both methods [128]. Another method for identifying muscles involved in dystonia may be fluorodeoxyglucose (FDG)-positron emission tomography (PET), which has been found useful in a case series containing 6 patients [129]. However, the low availability and high costs of FDG-PET are limiting factors.

Technological advances in the field of DBS are expected to revolutionize the treatment of hyperkinetic movement disorders. These include innovations in electrode design (*e.g.,* biomaterials providing less inflammation after implantation and electrodes with more than 8 stimulation contacts), in stimulation methods (*e.g.,* different stimulation waveforms or patterns), adaptive systems (*e.g.,* closed-loop stimulation), DBS imaging, and cranialization of the implantable pulse generator [125, 130].

As the invasiveness of DBS is an important limitation, non-invasive lesioning with high-intensity focused ultrasound (HIFU) and non-invasive stimulation (*e.g.*, transcranial magnetic stimulation or transcranial direct current stimulation) are of high investigational interest in the treatment of movement disorders. HIFU has been proven useful in Parkinson’s disease and essential tremor, albeit unilaterally and only in selected cases and selected indications [131, 132].

Randomized controlled trials - ideally comparing focused ultrasound and DBS - are, however, lacking, and availability is limited to absent in most countries. The use of HIFU in extrapyramidal hyperkinetic movement disorders is currently non-existent. Transcranial magnetic stimulation has been found effective in depression, and there is a possible future indication in dystonia and tic disorders [30].

In addition, research on different specific phenotypes of movement disorders is important because different phenotypes may need different treatment strategies. This may especially be the case in tardive syndromes. In the future, also, surgicogenomics and pharmacogenomics may be important to individualize treatment.

## CONCLUSION

This review offers an overview of all aspects regarding the treatment of extrapyramidal hyperkinetic movement disorders. Most disorders can be treated by multiple types of agents, which makes it difficult to see the forest for the trees. This review can be used as a guide in the stepwise treatment, but also in the management of adverse effects of different drugs. Next to medication, BoNT and DBS can be used in extrapyramidal hyperkinetic movement disorders. Mechanistic, pharmacological, and technological advances and well-designed RCTs are required to improve the treatment of extrapyramidal hyperkinetic movement disorders.

## Figures and Tables

**Table 1:** Management of main adverse effects of pharmacological therapies for extrapyramidal hyperkinetic movement disorders.

**Drug (Class)**	**Adverse Effect**	**Management**
DDAs	Parkinsonism	1. Dose reduction 2. * *<60 y:* anticholinergic * >60 y: amantadine 3. Levodopa
	Sedation	1. Dose reduction 2. Modafinil
	Depression	1. Dose reduction 2. Antidepressants
DRBAs	Sedation	1. Dose before sleep 2. Dose reduction 3. Switch to DRBA with lower risk
	Parkinsonism	1. Dose reduction 2. Switch to DRBA with lower risk 3. Anticholinergic agent
	Tardive dyskinesias	1. Dose reduction 2. Switch to atypical DRBA 3. Tetrabenazine
	Myoclonus	1. Dose reduction 2. Valproic acid
	Epilepsy	1. Dose reduction 2. Anti-epileptic drugs
	Neuroleptic malignant syndrome	1. Discontinuation 2. Supportive measures (cooling and hydration) 3. Dantrolene, bromocriptine, lorazepam 4. Electroconvulsive therapy
GABAergic drugs	Sedation (and other CNS adverse effects)	1. Dose holding 2. Dose reduction
	Withdrawal symptoms	1. Dose increase
Anticholinergics	PNS (*e.g.*, urinary retention, constipation, xerostomia)	1. Dose holding 2. Dose reduction 3. Treat specific symptoms
	CNS (*e.g.*, cognition, sedation)	1. Dose holding 2. Dose reduction
Amantadine	Confusion, impaired cognition (and other CNS adverse effects)	1. Dose reduction
	Livedo reticularis	1. Acceptation 2. Discontinuation
	Keratopathy	1. Discontinuation
Botulinum neurotoxin	Injection pain	1. Topical anesthetic agents
	Hematoma	1. Apply pressure or skin cooling
	Flu-like symptoms	1. Analgesics or antipyretic drugs 2. Switch brand

**Note:** Dose holding refers to keeping the dose unaltered for a considerable amount of time to allow the development of tolerance.

**Abbreviations**: CNS, central nervous system; DDAs, dopamine-depleting agents; DRBAs, dopamine receptor blocking agents; GABA, gamma-aminobutyric acid; PNS, peripheral nervous system.

**Table 2:** Suggested treatment initiation, titration and maintenance of most commonly used drugs for hyperkinetic movement disorders.

**Drug**	**Starting Dose**	**Titration** Increase with … Every Week*	**Common Therapeutic Dose**	**Maximum Dose**
Tetrabenazine	12.5 mg OD	12.5 mg	12.5-37.5 mg TID	100 mg TID
Haloperidol	0.5 mg OD	0.5 mg	1-5 mg BID	5 mg BID
Aripiprazole	10-15 mg OD	5 mg	15 mg OD	30 mg OD
Quetiapine	12.5 mg BID	25 mg	50-100 mg BID	400 mg BID
Clozapine	12.5 mg OD	12.5 mg	25-37.5 mg OD	100 mg OD
Clonazepam	0.25 mg OD	0.25 mg	0.5-1 mg BID	3 mg BID
Trihexyphenidyl^§^	2 mg TID	2 mg	2-5 mg TID	40 mg TID
Amantadine^‡^	100 mg OD	100 mg	100-200 mg BID	200 mg BID

**Abbreviations**: OD, once daily; BID, twice a day; mg, milligrams; TID; three times a day. *Symbols*: *, consider faster titration in case of high severity or medical emergency; §, consider slower treatment initiation in elderly or cognitively impaired individuals (*i.e.,* start with 1 mg OD, increase with 1 mg every week); ‡, no dosing in the afternoon or evening.

**Table 3:** Current clinical and investigational DBS targets for extrapyramidal hyperkinetic movement disorders.

**Movement Disorder**	**Setting**	
	***Routine Clinical *** ***Practice***	***Investigational***
Ballism	-	GPi
Chorea	-	GPi
Dystonia	GPi	STN
Tardive syndrome	GPi	-
Tics	-	Medial thalamus, GPi, IC-NcACC
Tremor (PD, dystonia)	VIM, PSA, DRT	-

**Abbreviations:** GPi, internal globus pallidus; IC, internal capsule; NcAcc, nucleus accumbens; PD, Parkinson’s disease; VIM, ventral intermediate nucleus of thalamus; PSA, posterior subthalamic area; DRT, dentatorubrothalamic tract; STN, subthalamic nucleus.

## LIST OF ABBREVIATIONS

ACh: Acetylcholine
ADHD: Attention-deficit Hyperactivity Disorder
AED: Antiepileptic Drugs
BoNT: Botulinum Neurotoxin
DBS: Deep Brain Stimulation
DDAs: Dopamine-depleting Agents
GABA: Gamma-aminobutyric Acid
GPi: Globus Pallidus Internus
OCD: Obsessive-compulsive Disorder
RCTs: Randomized Controlled Trials
