# Supplementary material for: Symptomatic Treatment of Extrapyramidal Hyperkinetic Movement Disorders
Source: Curr Neuropharmacol. 2024 May 20;22(14):2284–97. doi: 10.2174/1570159X22666240517161444 (PMC11451320; doi:10.2174/1570159X22666240517161444)
Supplement: Supplementary file 1 [file CN-22-2284_SD1.pdf]

Supplementary Material

Symptomatic Treatment of Extrapyramidal Hyperkinetic Movement Disorders

Gregory de Boer[1,2], Robertus Maria Alfonsius de Bie[1] and Bart Erik Kris Sylvain Swinnen[1,*]

[1]Department of Neurology and Clinical Neurophysiology, Amsterdam UMC Location University of Amsterdam, Meibergdreef 9, Amsterdam, The Netherlands; [2]Department of Neurology and Neurosurgery, University Medical Center Utrecht, Heidelberglaan 100, Utrecht, The Netherlands

Search Terms	Results (n Articles)
"movement disorders" [tiab] AND "treatment" [tiab]	256
"chorea" [title] AND "treatment" [tiab]	12
"ballism" [title] AND "treatment" [tiab]	1
"dystonia" [title] AND "treatment" [tiab]	107
"huntington's disease" [title] AND "treatment" [tiab]	910
"tardive" [title] AND "treatment" [tiab]	63
"tic disorders" [title] AND "treatment" [tiab]	35
"tourette syndrome" [title] AND "treatment" [tiab]	56
"movement disorders" [tiab] AND "botulinum" [tiab]	30
"dystonia" [title] AND "botulinum" [tiab]	65
"tardive" [title] AND "botulinum" [tiab]	1
"tic disorders" [title] AND "botulinum" [tiab]	1
"movement disorders" [tiab] AND "deep brain stimulation" [tiab]	58
"chorea" [title] AND "deep brain stimulation" [tiab]	2
"dystonia" [title] AND "deep brain stimulation" [tiab]	49
"huntington's disease" [title] AND "deep brain stimulation" [tiab]	3
"tardive" [title] AND "deep brain stimulation" [tiab]	5
"tic disorders" [title] AND "deep brain stimulation" [tiab]	2
"tourette syndrome" [title] AND "deep brain stimulation" [tiab]	15

1875-6190/24 $65.00+.00 **© 2024 Bentham Science Publishers**
